# Olink proteomics identifies FGF-19 as a treatment-responsive inflammatory biomarker associated with acupuncture intervention in young females with mild depression

**DOI:** 10.3389/fpsyt.2026.1743771

**Published:** 2026-02-19

**Authors:** Zhongxian Li, Zitong Jiao, Min Peng, Pan Zhang, Haiyan Xu, Ruiming Chen, Qiaoyu Ji, Jingjing Li, Lihua Chen, Beibei Zhao, Wenbin Fu, Peng Zhou

**Affiliations:** 1 Department of Acupuncture and Moxibustion, Shenzhen Bao'an District Traditional Chinese Medicine Hospital, Shenzhen, China; 2 The Seventh Clinical College of Guangzhou University of Chinese Medicine, Shenzhen, China; 3 Guangzhou University of Chinese Medicine, Guangzhou, China; 4 Department of Acupuncture and Moxibustion, The Second Affiliated Hospital of Guangzhou University of Traditional Chinese Medicine, Guangzhou, China

**Keywords:** acupuncture, FGF-19, inflammatory biomarkers, mild depression, olinkproteomics

## Abstract

Mild depression in women is a distinct disorder with unclear immune mechanisms. This study aims to identify peripheral inflammatory biomarkers and to explore acupuncture’s immunomodulatory effects via Olink proteomics. Thirty female participants (18–45 years) were assigned to healthy controls (HC), mild depression (MD), and acupuncture treatment (ACU). Plasma samples were analyzed using the Olink^®^ Target 96 Inflammation panel for differentially expressed proteins (DEPs) (Approval No. KY-2023-005-02), then analyzed for enrichment using Gene Ontology (GO) and Kyoto Encyclopedia of Genes and Genomes (KEGG). Correlations between DEPs and Hamilton Depression Rating Scale-17 items (HAMD-17) and Self-Rating Depression Scale (SDS) scores were assessed. Enzyme-linked immunosorbent assay (ELISA) was conducted on an expanded cohort (n=40/group) to validate key findings. Compared with HC, 15 proteins were significantly downregulated in MD (no proteins were upregulated), including FGF-19, SIRT2, and AXIN1. After acupuncture, six proteins were further downregulated, and three (FGF-19, FGF-5, IL-2) were significantly upregulated. ELISA validation confirmed that FGF-19, the sole protein significantly altered, was inversely correlated with depression scores and exhibited decreased expression in MD that was reversed by acupuncture. FGF-19’s bidirectional expression profile positions a promising inflammation-related biomarker and a potential treatment-responsive marker for mild depression in young females, also an indicator of acupuncture efficacy. Clinical Trial Registration Chinese Clinical Trial Registry, identifier ChiCTR2300068054.

**Clinical Trial Registration:**

https://www.chictr.org.cn/showprojEN.html?proj=189355, identifier ChiCTR2300068054.

## Graphical Abstract

**Figure:**
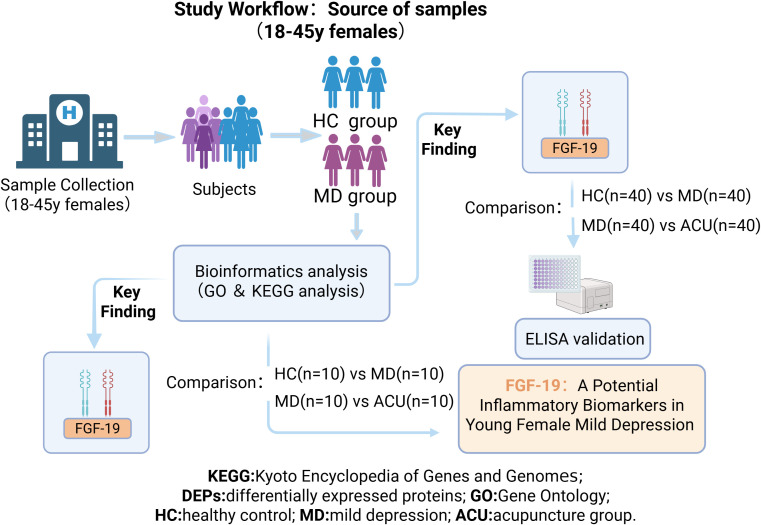

## 1 Introduction

Depression is one of the most common psychiatric disorders worldwide, affecting more than 300 million individuals and accounting for approximately 4.4% of the global population (1). The global prevalence of depression has increased substantially in recent years, partly driven by large-scale public health crises such as the Coronavirus Disease 2019 (COVID-19) pandemic. In 2020 alone, an estimated 53.5 million new cases of depression were reported globally, representing a 27.6% increase compared with 2019 (2). According to the Global Burden of Disease studies, both the prevalence and disease burden of depressive disorders are consistently higher in women than in men, with the burden in women estimated to be up to 1.6 times greater (3). This sex disparity is also evident in China, where lifetime and 12-month prevalence rates of depression are higher among women, particularly during hormonally sensitive periods such as the reproductive years and menopause. Despite expanding treatment options, overall service coverage for depression remains suboptimal, especially among women (4).

Accumulating evidence suggests that immune and inflammatory processes play an important role in the pathophysiology of depression. Peripheral inflammatory mediators, including C-reactive protein (CRP), interleukin-6 (IL-6), and interleukin-1 receptor antagonist (IL-1RA), have been associated with depressive symptoms and may influence central neurotransmission and neuroplasticity through neuroendocrine–immune signaling and microglial activation (5–7). In addition, inflammatory conditions are associated with an increased risk of depressive symptoms, and anti-inflammatory interventions have shown beneficial effects in specific subgroups of patients, supporting the notion that immune dysregulation contributes to depression in at least a subset of individuals (8–11).

Acupuncture, a core therapeutic modality in traditional Chinese medicine, has attracted increasing attention as an adjunctive intervention for depression (12). Previous studies suggest that acupuncture may exert antidepressant effects by modulating neuroendocrine–immune interactions, enhancing neuroplasticity, and reducing pro-inflammatory signaling (13–15). However, most existing studies have focused on a limited number of predefined biomarkers, and systematic, high-throughput investigations into the molecular correlates of acupuncture—particularly in women with mild depression—remain scarce.

Given the heterogeneity of depression and the complexity of immune–neural crosstalk, system-level profiling of peripheral inflammatory proteins may provide a more comprehensive view of coordinated molecular alterations and help identify treatment-associated signatures. The Olink^®^ proximity extension assay (PEA) platform is a high-throughput proteomic technology that enables simultaneous quantification of multiple inflammation-related proteins with high sensitivity and minimal sample requirements. This platform has been increasingly applied in psychiatric research, including studies of bipolar disorder and schizophrenia (16–18), but has not yet been systematically used to evaluate peripheral immune changes associated with acupuncture in women with mild depression.

Building upon our group’s prior clinical research in central nervous system imaging (ChiCTR2300068054) (19), the present study adopts a central–peripheral immune perspective to explore peripheral inflammatory protein alterations in young females with mild depression using the Olink PEA platform. The aims of this study were to identify inflammation-related protein signatures associated with mild depression in women and to explore treatment-associated changes following acupuncture intervention. By integrating proteomic profiling with clinical assessments, this exploratory study seeks to provide new insights into peripheral immune alterations related to female mild depression.

## 2 Materials and methods

**Figure 1:**
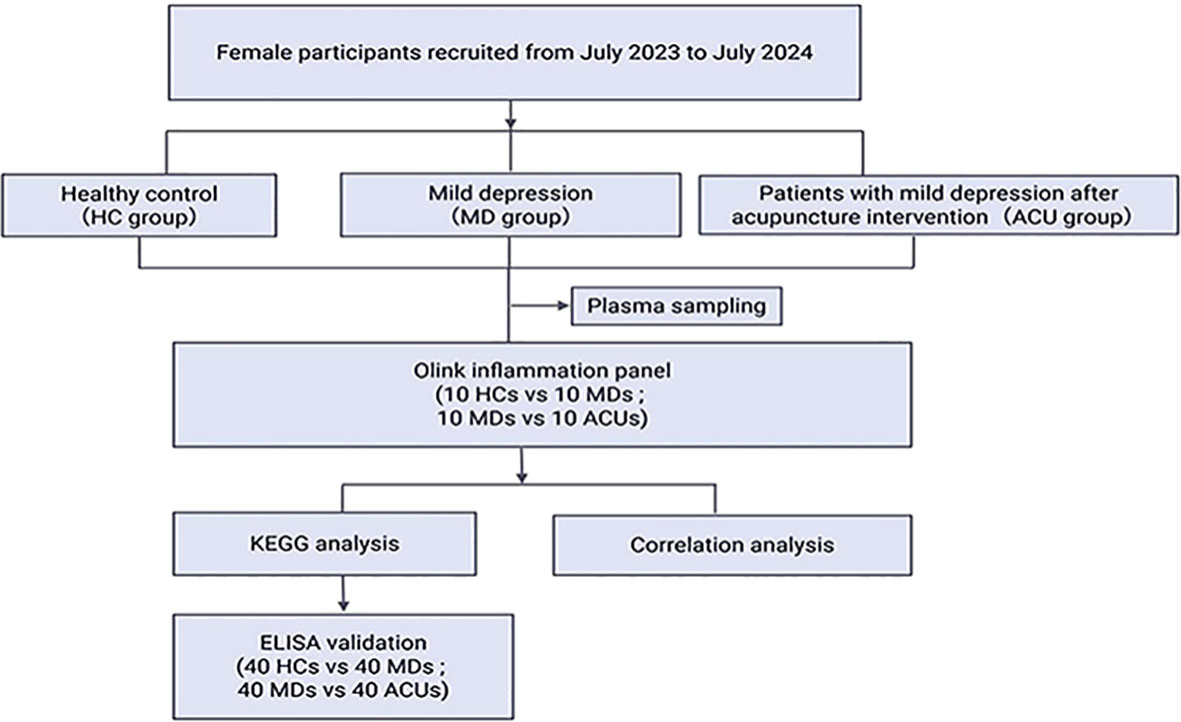
Overview of the clinical trial and Olink proteomics workflow for evaluating acupuncture in mild depression.

The workflow of the present study is illustrated in **Figure 1**.

### 2.1 Ethics statement

All study procedures were reviewed and approved by the Ethics Committee of Bao’an District Hospital of Traditional Chinese Medicine, Shenzhen, China (Approval No. KY-2023-005-02). The trial was registered with the Chinese Clinical Trial Registry (Registration No. ChiCTR2300068054). All procedures were conducted in accordance with the Declaration of Helsinki. Written informed consent was obtained from all participants prior to enrollment.

### 2.2 Participants

**Figure 2:**
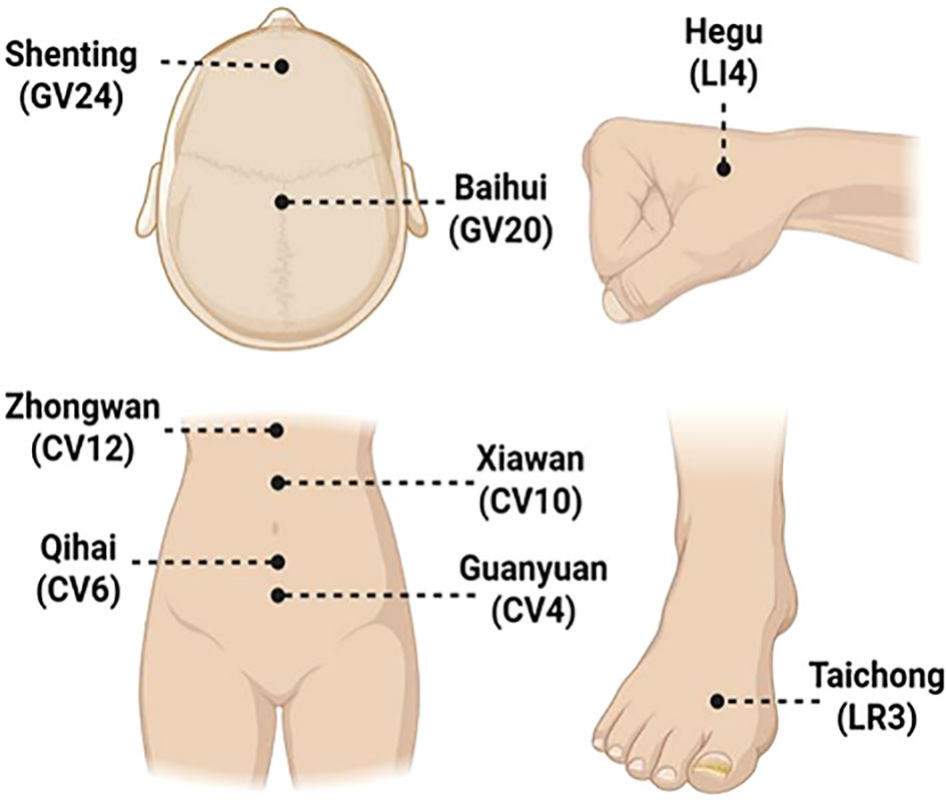
Anatomical locations of the selected acupoints used in acupuncture therapy.

All participants were recruited from Bao’an District Hospital of Traditional Chinese Medicine, Shenzhen, China, between July 26, 2023 and July 25, 2024. Female patients aged 18 to 45 years were included if they met the diagnostic criteria for mild depression according to International Classification of Diseases, 10th revision (ICD-10), with a HAMD-17 score >7 and <17, and an SDS score ≥53. Exclusion criteria were: (1) a history of suicidal ideation, schizophrenia, bipolar disorder, or other severe psychiatric disorders; (2) comorbid conditions such as infectious, cardiovascular, or respiratory diseases, substance abuse, or alcohol dependence; (3) fear of or intolerance to acupuncture; (4) use of antidepressants or other psychological interventions within the previous six weeks; (5) any immunological or inflammatory disorders or use of anti-inflammatory or immunosuppressive agents; and (6) pregnancy, lactation, or attempting to conceive. After skin disinfection, eligible patients received standardized acupuncture treatment at Baihui (GV20), Shenting (GV24), Zhongwan (CV12), Xiawan (CV10), Qihai (CV6), Guanyuan (CV4), Hegu (LI4), and Taichong (LR3) by licensed acupuncturists with at least 5 years of experience in the clinic, with Acupuncture needles in 0.25×25 mm and 0.25×40 mm sizes(Huatuo, Suzhou, China). (acupoint selection was performed according to the standardized anatomical locations published by the World Health Organization (WHO) for the Western Pacific Region, while needle manipulation followed the technical guidelines outlined in a nationally recognized Chinese acupuncture textbook). After needle insertion, both twirling-rotating and lifting-thrusting techniques are applied with uniform intensity to elicit deqi. Each session lasted 30 minutes, three times per week for six consecutive weeks. Acupuncture therapy was performed by licensed acupuncturists with at least 5 years of experience in the clinic. All participants received no other medication or physical therapy for depression during the acupuncture intervention period of this study. Specific details and the subsequent management process of puncture-related adverse events will be accurately documented. No serious acupuncture-related adverse events were observed during the intervention period. Details of the acupuncture protocol are provided in **Table 1** and **Figure 2**.

**Table 1:** Treatment parameters.

Serial number	Acupuncture intervention	Details
1	Acupoints	Baihui(GV20), Shenting(GV24), Zhongwan(CV12), Xiawan(CV10), Qihai(CV6), Guanyuan(CV4), Hegu(LI4), Taichong(LR3)
2	Needle type	0.25×25 mm (Huatuo, Suzhou, China), 0.25×40 mm (Huatuo, Suzhou, China)
3	Depth description	GV20 and GV24 were inserted obliquely to a depth of 10mm; LI4 and LR3 were inserted perpendicularly to a depth of 15mm; CV12, CV10, CV6, and CV4 were inserted perpendicularly to a depth of 20mm
4	Needle stimulation	After achieving the desired depth, needle manipulation (lifting-thrusting and twirling-rotating) was performed until “deqi” (sensations of soreness, numbness, distention, heaviness) sensation was elicited
5	Needle retention time	30 mins
6	Frequency and duration of treatment sessions	Three times a week for 6 weeks
7	Operator qualification	Acupuncture therapy was performed by licensed acupuncturists with at least 5 years of experience in the clinic
8	Other interventions	All participants received no other medication or physical therapy for depression during the acupuncture intervention period of this study

In parallel, age- and BMI-matched healthy female volunteers were recruited as controls. Inclusion criteria for the control group were: (1) matched baseline characteristics (e.g., age and BMI) with the acupuncture group; (2) a HAMD-17 score <8 and SDS score <53; (3) no personal or family history of psychiatric disorders; (4) no major chronic or infectious diseases; and (5) normal inflammatory markers during screening. All participants provided written informed consent before enrollment.

### 2.3 Plasma sample collection and storage

Peripheral venous blood (2-4mL) was collected from each participant under fasting conditions using EDTA-coated anticoagulant tubes. Immediately after collection, the tubes were gently inverted several times to prevent clotting. The blood samples were left at room temperature for 15 to 30 minutes, followed by centrifugation at 3000 rpm for 10 minutes to separate the plasma. The upper clear plasma layer was carefully aspirated, avoiding any disturbance of the cellular layer or pellet. Plasma was aliquoted into sterile cryovials (200 µL per tube), labeled with sample ID, collection date, and participant information, and immediately stored at –80°C to ensure long-term stability and analytical reliability. Blood samples were collected outside the menstrual period. Women who were pregnant, preparing for pregnancy, or breastfeeding were excluded.

### 2.4 Inflammation-related biomarkers analysis

Plasma samples from female healthy controls (n=10) and female patients with mild depression (n=10), collected before and after acupuncture treatment, were analyzed using the Olink^®^ Target 96 Inflammation panel. This platform is based on the high-specificity and multiplex PEA technology, which enables simultaneous detection of 92 protein biomarkers per sample. In brief, each target protein is recognized by a pair of matched antibodies, each conjugated to a unique DNA oligonucleotide. Upon binding to the same target, the oligonucleotides are brought into proximity and hybridize, forming a double-stranded DNA “barcode” that serves as a template for DNA polymerase–mediated extension. The resulting DNA is then amplified using real-time quantitative PCR (qPCR) performed on a microfluidic platform. Fluorescence signals corresponding to each protein are transformed into Normalized Protein Expression (NPX) values after log2 transformation. NPX is an arbitrary unit on a log2 scale, allowing relative quantification of protein expression between samples.

### 2.5 Bioinformatics analysis

DEPs from the Olink data were identified using the limma package in R. Nominal p-values were used for exploratory screening (p < 0.05), and Benjamini–Hochberg FDR-adjusted p-values were additionally calculated and reported as a reference.

### 2.6 ELISA validation

The plasma expression levels of FGF-19 were further validated using a commercially available ELISA kit (Shenzhen Xinbosheng Biotechnology Co., Ltd., China, *catalogue number: H241230-146a*) according to the manufacturer’s instructions. A total of 120 samples were analyzed, including plasma from female healthy controls (n=40) and female patients with mild depression (n=40) before and after acupuncture treatment. Each sample was assayed in duplicate, and optical density (OD) values were measured using a microplate reader. The final concentrations were calculated based on the standard curve provided with the kit.

### 2.7 Statistical analysis

All data are presented as mean ± standard deviation (SD), unless otherwise specified. Statistical analyses were performed using IBM SPSS Statistics version 24.0 and R software version 4.1.3. Normality was assessed using the Shapiro–Wilk test. For normally distributed variables, differences between independent groups (HC vs MD) were assessed using independent-sample t-tests, and within-group comparisons (MD vs ACU) were evaluated using paired-sample t-tests. For non-normally distributed data, the Mann–Whitney U test was used for between-group comparisons, and the Wilcoxon signed-rank test was applied for within-group comparisons.

For Olink proteomic data, NPX values (log2 scale) were used as input. Differential protein expression was assessed using the limma package in R, reporting log fold changes (logFC) and nominal p-values. In addition, multiple-testing adjusted p-values were calculated using the Benjamini–Hochberg procedure (FDR-adjusted p-values) and provided as a reference/sensitivity assessment. Given the exploratory nature of this targeted proteomics study, candidate differentially expressed proteins (DEPs) were primarily screened using nominal p < 0.05 without applying a predefined fixed |logFC| cutoff, and key findings were further validated in an expanded cohort by ELISA.

Correlation analyses between protein levels and clinical scale scores (HAMD-17 and SDS) were performed using Pearson correlation coefficients (or Spearman correlation when normality assumptions were not met). ROC curve analyses were conducted in R to evaluate discriminative performance, and AUC values with 95% confidence intervals were reported. A two-sided p-value < 0.05 was considered statistically significant.

## 3 Results

### 3.1 Clinical characteristics of the participants

A total of 40 female patients with mild depression and 40 age- and sex-matched healthy controls were enrolled in the study. For each patient, peripheral plasma samples were collected both before and after a 6-week course of acupuncture treatment, resulting in a total of 120 plasma samples. The healthy control group is referred to as the HC group, the pre-treatment patient group as the MD group, and the post-treatment group as the ACU group.

Baseline characteristics were compared between the HC and MD groups. No significant differences were found in age or BMI (*P*>0.05), indicating that the groups were comparable. However, the MD group had significantly higher HAMD-17 and SDS scores than the HC group (both *P* < 0.05), confirming the presence of mild depressive symptoms at baseline. After acupuncture treatment, both HAMD-17 and SDS scores in the ACU group were significantly reduced compared to pre-treatment levels (*P* < 0.05), as shown in **Table 2**.

**Table 2:** Baseline demographics and clinical characteristics of included participants.

Baseline characteristics	Cohorts for Olink inflammatory assay				Cohorts for ELISA validation			
	HC group	MD group	ACU group	*P*	HC group	MD group	ACU group	*P*
Sample Size	N=10	N=10	N=10		N=40	N=40	N=40	
Age(years)	27.60 ± 4.45	28.10 ± 3.87		0.792	34.85 ± 6.85	33.38 ± 6.55		0.328
BMI(kg/m^2^)	20.67 ± 1.21	20.49 ± 1.11		0.729	21.36 ± 1.54	21.75 ± 1.45		0.246
HAMD-17	3.20 ± 1.32	13.70 ± 1.49^△^	5.30 ± 2.21^※^	0.000	3.43 ± 1.78	14.10 ± 1.93^△^	4.73 ± 2.03^※^	0.000
SDS	43.90 ± 3.90	58.10 ± 1.97^△^	48.70 ± 2.91^※^	0.000	41.73 ± 4.32	58.23 ± 2.54^△^	47.80 ± 2.78^※^	0.000

Data are presented as mean ± SD. Normality was assessed using the Shapiro–Wilk test. ^△^indicates a significant difference compared to the HC group (*P* < 0.01; independent-sample t-test or Mann–Whitney U test); ^※^indicates a significant difference compared to the MD group (*P* < 0.01; paired-sample t-test or Wilcoxon signed-rank test).

### 3.2 Potential biomarkers screening

**Figure 3:**
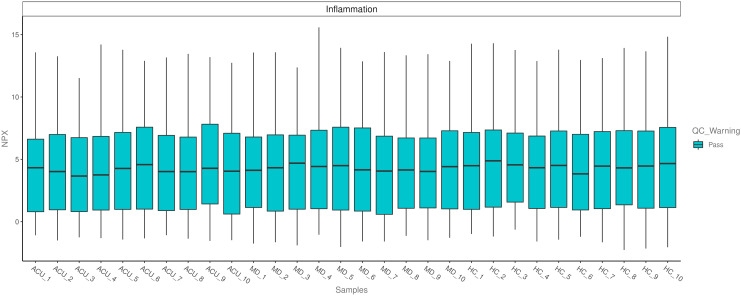
Quality control assessment of Olink inflammation panel data across all samples. Note: Box plots are shown for each sample, with the x-axis representing sample identifiers and the y-axis indicating NPX values. Each box plot displays five statistical measures: maximum, upper quartile (Q3), median, lower quartile (Q1), and minimum.

**Figure 4:**
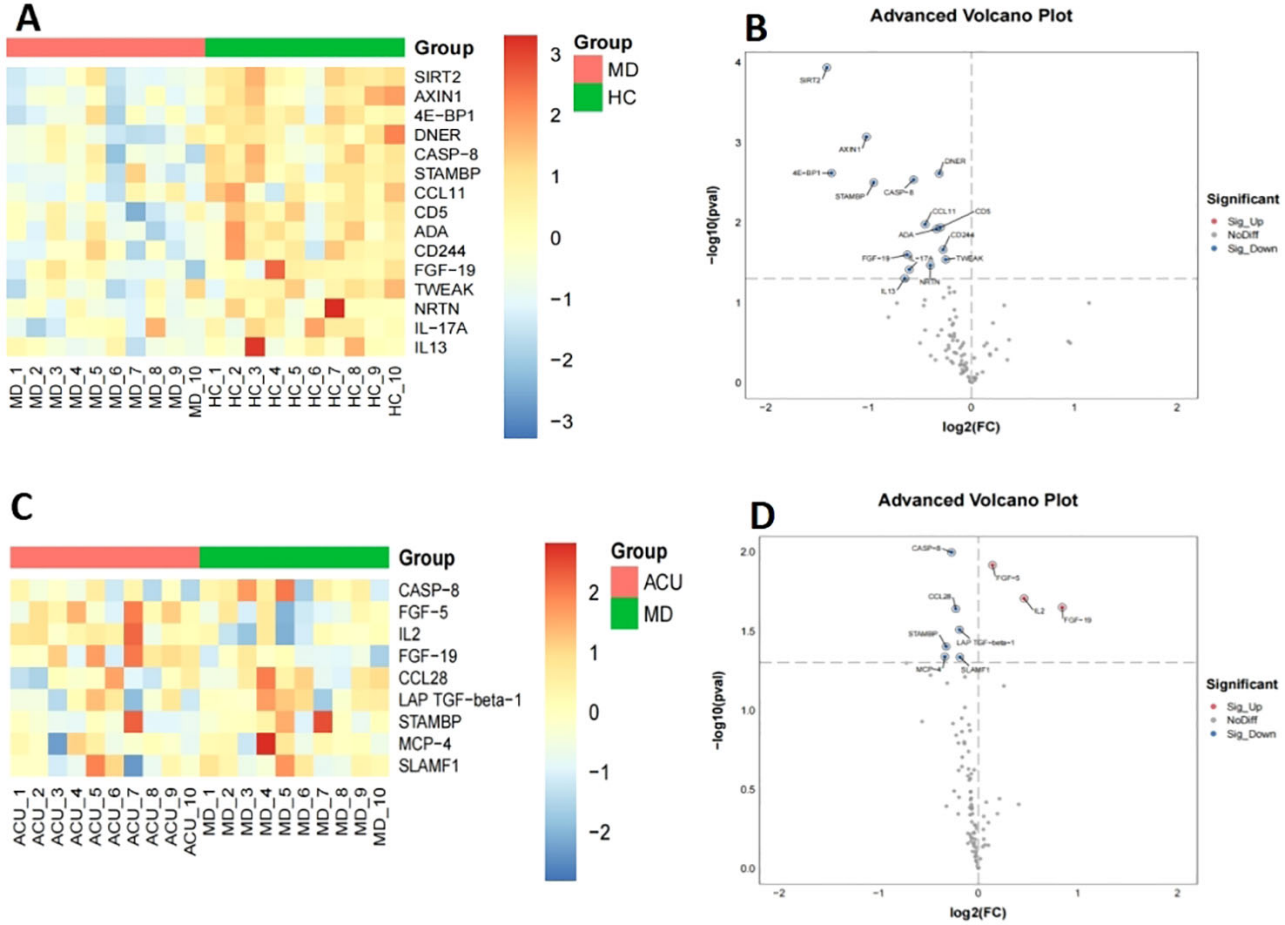
Expression profiles of DEPs in pairwise group comparisons. **(A)** Heatmap of 15 significantly downregulated DEPs identified between HC and MD groups; **(B)** Volcano plot of 92 inflammation-related proteins comparing HC vs MD. Red: significantly upregulated proteins; Blue: significantly downregulated proteins; Gray: not significant; **(C)** Heatmap of 9 DEPs identified between MD and ACU groups after acupuncture intervention; **(D)** Volcano plot of 92 inflammation-related proteins comparing MD vs ACU. Color coding as in panel **(B)**.

A total of 30 plasma samples from three groups were initially subjected to internal quality control using the Olink platform. The results demonstrated good reproducibility and no significant technical deviations among samples, meeting the requirements for downstream analysis (**Figure 3**). Differential expression analysis of 92 inflammation-related proteins was subsequently performed across groups. Compared with the HC group, 15 proteins were significantly downregulated in the MD group, including FGF-19, SIRT2, AXIN1, 4E-BP1, DNER, CASP-8, STAMBP, CCL11, CD5, ADA, CD244, TWEAK, NRTN, IL-17A, and IL-13 (**Figures 4A, B**). When comparing the MD and ACU groups, six proteins showed further downregulation following acupuncture treatment (CASP-8, CCL28, LAP TGF-beta-1, STAMBP, MCP-4, and SLAMF1), while three proteins (FGF-19, FGF-5 and IL-2) were significantly upregulated (**Figures 4C, D**).

**Figure 5:**
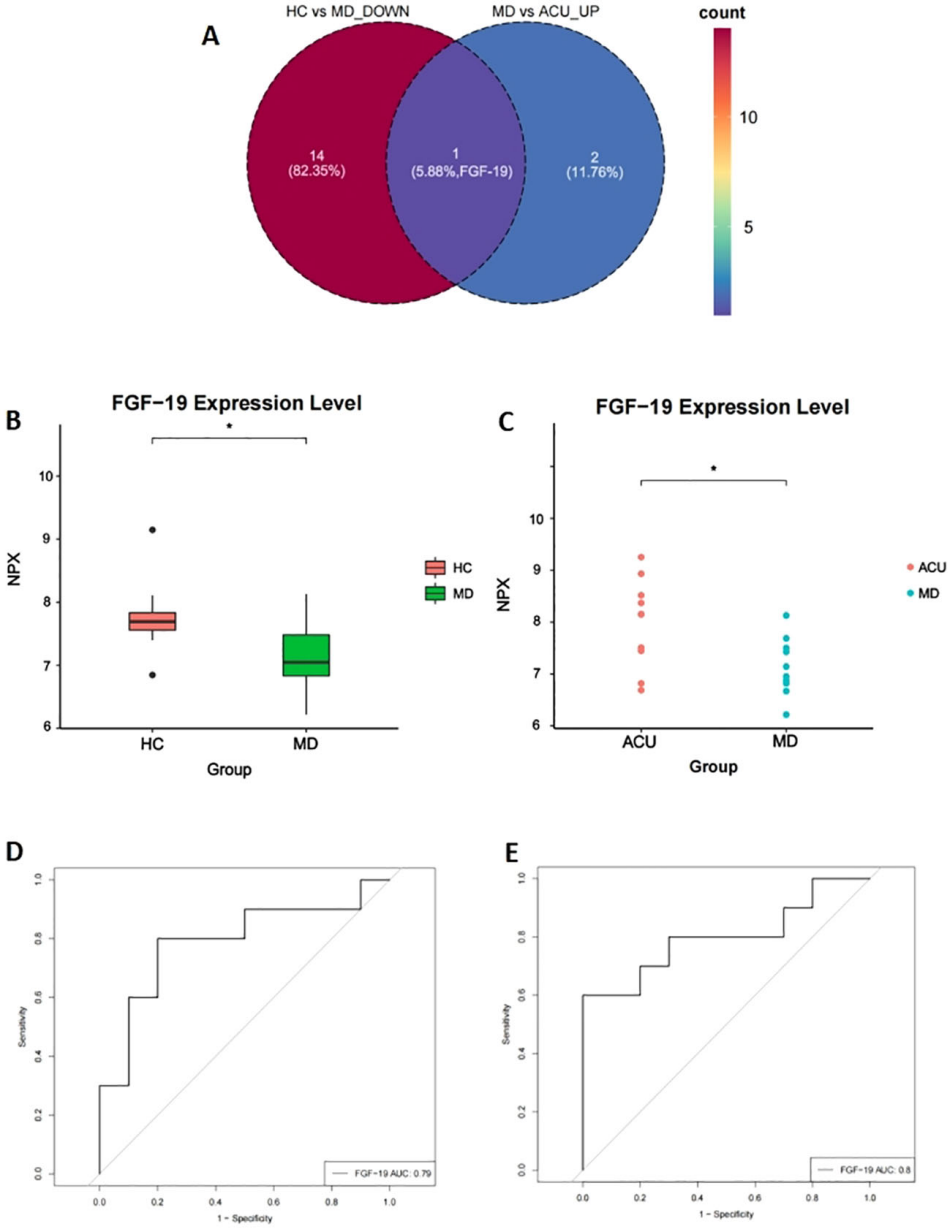
Dynamic expression pattern and diagnostic performance of FGF-19 across groups. **(A)** Venn diagram showing DEPs with opposite expression trends in HC vs MD (downregulated) and MD vs ACU (upregulated) comparisons; only FGF-19 was shared between both contrasts; **(B)** Comparison of FGF-19 expression levels between HC and MD groups; **(C)** Comparison of FGF-19 expression levels between MD and ACU groups; **(D)** ROC curve evaluating the diagnostic performance of FGF-19 in distinguishing MD from HC; **(E)** ROC curve evaluating the diagnostic performance of FGF-19 in distinguishing ACU from MD. * P < 0.05.

At baseline, plasma FGF-19 levels were negatively correlated with HAMD-17 scores in patients with mild depression, indicating that lower FGF-19 levels were associated with greater symptom severity. After acupuncture treatment, the relationship between FGF-19 levels and HAMD-17 scores differed from baseline, which may reflect concurrent changes in biomarker levels and symptom distributions following treatment rather than a stable or direct linear association (**Figures 5A–C**). To further evaluate the discriminatory performance of FGF-19, ROC curve analysis was conducted. The AUC for FGF-19 in distinguishing MD from HC was 0.79 (95% CI: 0.55–0.98) (**Figure 5D**). Similarly, the AUC for differentiating pre- and post-acupuncture states (MD vs ACU) was 0.80 (95% CI: 0.57–0.98) (**Figure 5E**). These AUC values indicate a moderate discriminative ability of FGF-19 for distinguishing disease status and treatment-associated changes at the group level.

### 3.3 Functional enrichment analysis of DEPs

To explore the potential biological roles of the DEPs, GO and KEGG enrichment analyses were performed.

**Figure 6:**
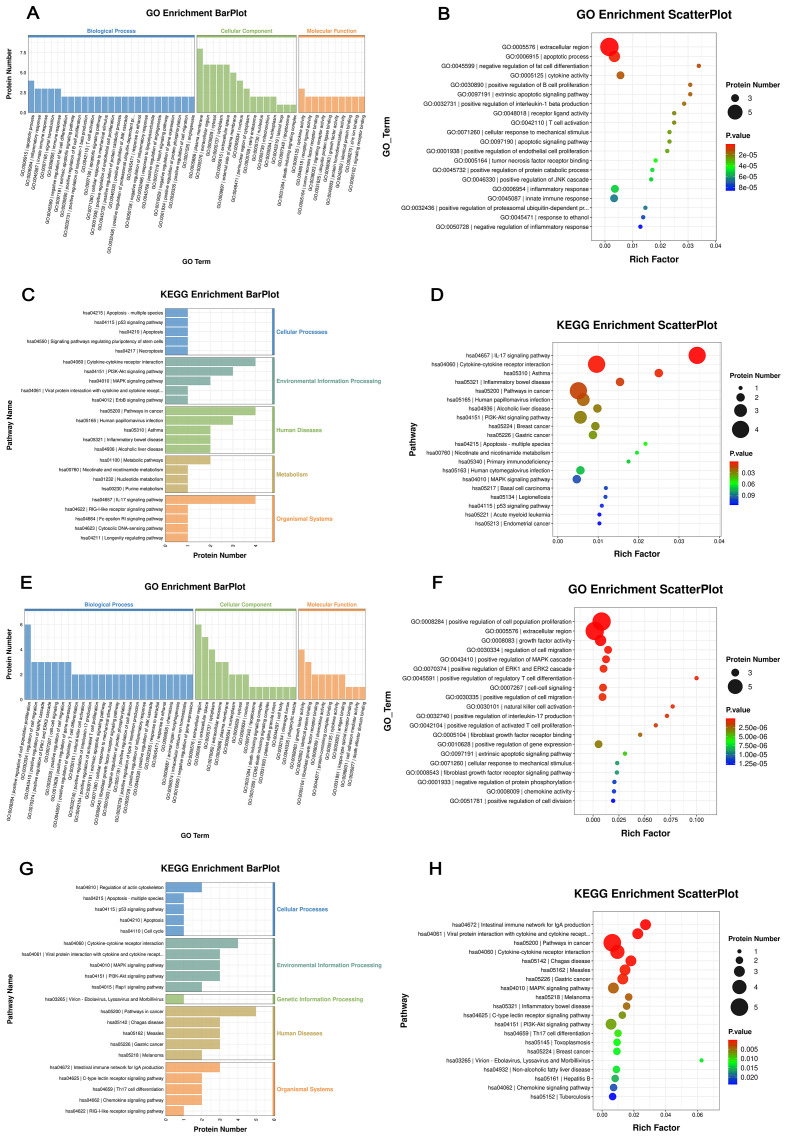
Functional enrichment analysis of DEPs between groups. **(A–D)** DEPs between healthy controls (HC) and patients with mild depression (MD); **(E–H)** DEPs identified before and after acupuncture treatment (MD vs ACU group). **(A, E)** GO enrichment bar plots based on all annotated proteins, showing enrichment in three categories: BP, CC, and MF; **(B, F)** GO enrichment scatter plots based on the 92 inflammation-related protein background, displaying rich factor, protein count, and P-values; **(C, G)** KEGG pathway enrichment bar plots based on all annotated proteins; **(D, H)** KEGG pathway enrichment scatter plots based on the 92 inflammation-related protein background, highlighting significantly enriched signaling pathways.

For the comparison between the HC and MD groups, GO analysis revealed that the DEPs (n=15) were primarily enriched in apoptotic process, inflammatory response, and innate immune response in the biological process (BP) category. In terms of cellular component (CC), these DEPs were mainly located in the plasma membrane, extracellular region, and cytosol. Regarding molecular function (MF), the proteins were significantly enriched in cytokine activity, receptor ligand activity, and TNF receptor binding (**Figures 6A, B**). KEGG pathway analysis indicated that these DEPs were predominantly involved in cytokine–cytokine receptor interaction, the PI3K-Akt signaling pathway, and pathways in cancer (**Figures 6C, D**).

In the comparison between the MD and ACU groups, GO analysis showed that the nine identified DEPs were enriched in biological processes such as positive regulation of cell population proliferation, regulation of cell migration, and positive regulation of MAPK cascade. The cellular component analysis indicated enrichment in the extracellular region, extracellular space, and cytoplasm. For molecular function, the DEPs were mainly enriched in growth factor activity, identical protein binding, and fibroblast growth factor receptor binding (**Figures 6E, F**). Consistently, KEGG pathway analysis demonstrated that these DEPs were also involved in cytokine–cytokine receptor interaction, PI3K-Akt signaling pathway, and pathways in cancer (**Figures 6G, H**).

### 3.4 Correlation between DEPs and clinical features

**Figure 7:**
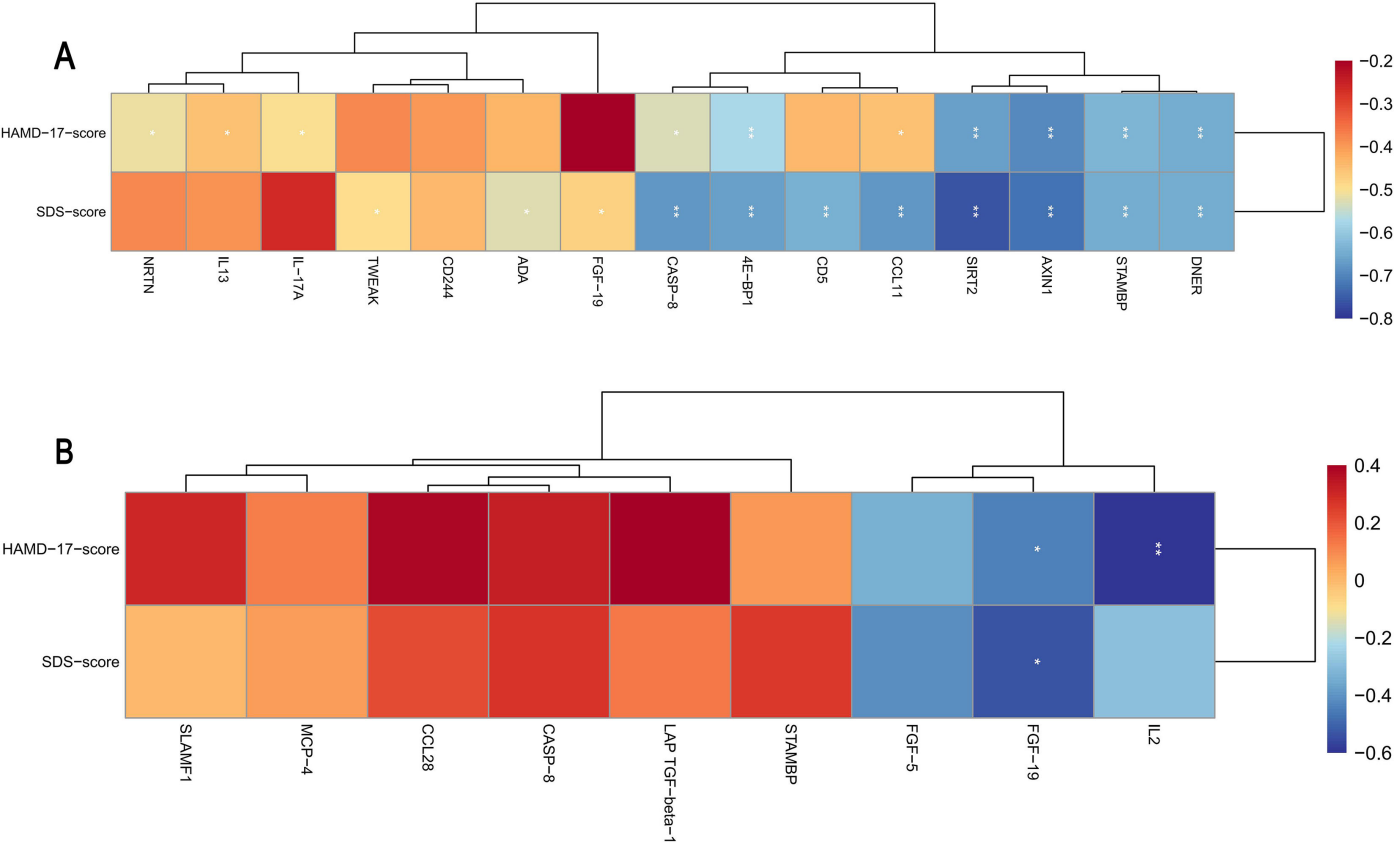
Correlation analysis between DEPs and depression rating scale scores. **(A)** Heatmap showing the correlation between DEPs and HAMD-17 and SDS scores in the comparison between HC and MD groups; **(B)** Correlation heatmap between DEPs and HAMD-17 and SDS scores after acupuncture (MD vs ACU groups); Statistical significance: *P < 0.05, **P < 0.01.

Pearson correlation analysis was performed between the expression levels of inflammation-related DEPs and clinical depression scores (HAMD-17 and SDS). In the comparison between HC and MD groups, AXIN1, SIRT2, DNER, STAMBP, 4E-BP1, CASP-8, NRTN, IL-17A, CCL11, and IL-13 were all significantly negatively correlated with HAMD-17 scores (*P* < 0.05). In addition, FGF-19, SIRT2, AXIN1, CASP-8, CCL11, 4E-BP1, STAMBP, DNER, CD5, ADA, and TWEAK were significantly negatively correlated with SDS scores (*P* < 0.05) (**Figure 7A**).

In the comparison between MD and ACU groups (i.e., pre- and post-acupuncture), FGF-19 and IL-2 expression levels were significantly positively correlated with HAMD-17 scores (*P* < 0.05), and FGF-19 was also positively correlated with SDS scores (*P* < 0.05) (**Figure 7B**). This post-treatment correlation was observed within the post-acupuncture group and may reflect residual inter-individual variability following intervention.

### 3.5 Elisa validation

**Figure 8:**
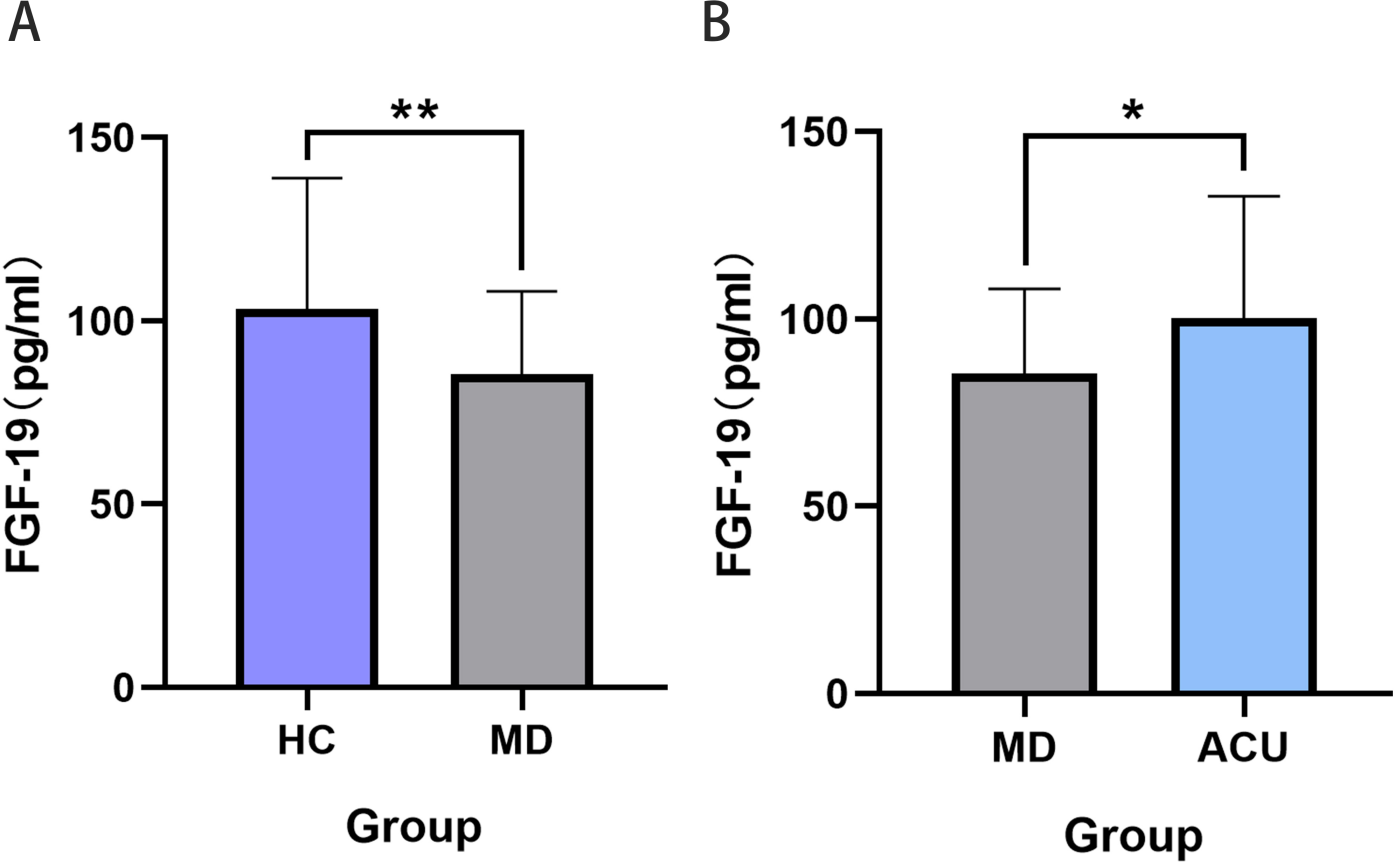
ELISA validation of FGF-19 expression levels among groups. **(A)** FGF-19 levels were significantly decreased in the MD group compared to the HC group; **(B)** FGF-19 levels were significantly increased after acupuncture intervention (MD group vs ACU group). Statistical significance: *P < 0.05, **P < 0.01.

To further validate the clinical relevance of FGF-19, an expanded cohort of 40 participants per group was analyzed using ELISA. The results showed that FGF-19 expression was significantly lower in the MD group compared to the HC group (*P* < 0.001), and was significantly increased in the ACU group following acupuncture treatment compared to the MD group (*P* < 0.01). These findings were consistent with those obtained from the Olink proteomic analysis (**Figure 8**).

## 4 Discussion

In this exploratory study, we identified fibroblast growth factor 19 (FGF-19) as a candidate inflammation-related biomarker associated with mild depression in young females. At baseline, plasma FGF-19 levels were inversely correlated with depressive symptom severity, suggesting that reduced FGF-19 expression may be linked to greater symptom burden in the depressive state. Following acupuncture intervention, FGF-19 levels increased in parallel with overall symptom improvement, indicating that this molecule may also reflect treatment-associated biological changes.

Notably, the relationship between FGF-19 levels and clinical symptoms appeared to differ between baseline and post-treatment states. This observation suggests that FGF-19 may carry distinct biological information depending on the underlying clinical context. In the depressive state, lower FGF-19 levels may be associated with disease-related immune or metabolic alterations, whereas after intervention, changes in FGF-19 may reflect a broader shift toward a regulated or compensated biological state. Such context-dependent associations are consistent with the notion that peripheral biomarkers do not necessarily exhibit fixed linear relationships across different disease or treatment phases.

Most previous studies have focused on elevated inflammatory markers, such as CRP, IL-1RA, and CCL2, particularly in patients with moderate-to-severe depression (9). In contrast, the present study observed a downward trend in FGF-19 and several other immune-related proteins in young females with mild depression. This pattern may reflect differences in symptom severity, disease stage, or demographic characteristics, and suggests that mild depression could be associated with a distinct peripheral immune profile. These findings highlight the potential heterogeneity of immune alterations across depressive subtypes and underscore the importance of stratified biomarker investigations.

FGF-19 is best characterized as a regulator of bile acid metabolism and energy homeostasis through FXR-dependent signaling pathways. Increasing evidence indicates that bile acid signaling and FXR activity are closely linked to inflammatory regulation and neurobehavioral processes. From this perspective, reduced FGF-19 expression may be associated with altered peripheral immune–metabolic balance, which could indirectly influence mood-related processes through gut–brain or immune–neural interactions (20–22).

Pathway enrichment analysis further suggested that FGF-19 and other altered proteins were associated with inflammation-related signaling pathways, including PI3K-Akt, MAPK/ERK, NF-κB, and NLRP3 (23, 24). In addition to FGF-19, several immune-regulatory proteins—such as SIRT2, AXIN1, CASP-8, and STAMBP—were also downregulated in patients with mild depression, potentially reflecting early or coordinated immune dysregulation. Although these proteins did not show significant recovery following acupuncture, their shared involvement in inflammatory, metabolic, and neuroplastic pathways suggests that mild depression may involve interconnected peripheral signaling changes rather than isolated molecular abnormalities (25).

Taken together, these findings position FGF-19 as a dynamic, state-associated biomarker that may reflect both depressive status and treatment-associated biological shifts at the group level. While mechanistic validation is required, the observed patterns support the value of system-level proteomic profiling for identifying candidate immune markers relevant to female mild depression and its modulation by acupuncture.

### 5.1 Limitations

Several limitations of this study should be acknowledged. First, although the sample size was expanded for ELISA validation, the overall cohort size remains relatively limited, which may restrict the generalizability of the findings. In addition, the study population was restricted to young females, which reduced heterogeneity but limits extrapolation to males or other age groups. Although age and sex restriction helped control major sources of variability, menstrual cycle phase and exogenous hormone use were not systematically recorded, and residual hormonal influences on inflammatory protein expression cannot be fully excluded.

Second, the absence of a sham acupuncture or waiting-list control group precludes definitive attribution of the observed changes in FGF-19 to acupuncture-specific effects. Accordingly, the treatment-associated alterations observed in this study should not be interpreted as evidence of causal or mechanism-specific efficacy.

Third, selection bias may have been introduced during the validation phase due to resource constraints that necessitated prioritization strategies. Candidate proteins were selected based on predefined criteria, including intergroup differences and correlations with clinical outcomes. While this approach facilitated efficient validation of core hypotheses, it may have overlooked biologically relevant proteins with smaller effect sizes or indirect clinical associations.

Finally, this study is exploratory in nature and primarily aimed at identifying candidate biomarkers through high-throughput screening. Although pathway enrichment analysis provided supportive biological context, no functional experiments were conducted to directly assess causal mechanisms. Future studies incorporating larger cohorts, sham-controlled clinical designs, and multi-level central–peripheral immune–neural analyses will be necessary to clarify the mechanistic role of FGF-19 and its relevance to acupuncture-associated effects.

## 6 Conclusion

In this preliminary exploratory study, FGF-19 was identified as a candidate inflammation-related biomarker associated with mild depression in young females. Its decreased expression in depression and treatment-associated increase following acupuncture were supported by proteomic profiling and ELISA validation. These findings suggest that FGF-19 may have potential value as a state-associated and treatment-responsive marker at the group level. Further studies in larger, well-controlled cohorts and mechanistic models are required to validate these observations and to clarify the role of peripheral immune alterations in depression and acupuncture-associated effects.

## Data Availability

The datasets presented in this study can be found in online repositories. The names of the repository/repositories and accession number(s) can be found in the article/**Supplementary Material**.

## References

[1] World Health Organization . Depression and Other Common Mental Disorders: Global Health Estimates. Geneva: World Health Organization (2017).

[2] COVID-19 Mental Disorders Collaborators . Global Prevalence and Burden of Depressive and Anxiety Disorders in 204 Countries and Territories in 2020 Due to the COVID-19 Pandemic. Lancet. (2021) 398:1700–12. doi: 10.1016/S0140-6736(21)02143-7, PMID: 34634250 PMC8500697

[3] GBD 2019 Diseases and Injuries Collaborators . Global Burden of 369 Diseases and Injuries in 204 Countries and Territories, 1990–2019: A Systematic Analysis for the Global Burden of Disease Study 2019. Lancet. (2020) 396:1204–22. doi: 10.1016/S0140-6736(20)30925-9, PMID: 33069326 PMC7567026

[4] Lu J Xu X Huang Y Li X Xu W Li Y . Prevalence of Depressive Disorders and Treatment in China: A Cross-Sectional Epidemiological Study. Lancet Psychiatry. (2021) 8:981–90. doi: 10.1016/S2215-0366(21)00251-0, PMID: 34559991

[5] Slavich GM Irwin MR . From stress to inflammation and major depressive disorder: a social signal transduction theory of depression. Psychol Bull. (2014) 140:774–815. doi: 10.1037/a0035302, PMID: 24417575 PMC4006295

[6] Smit AJT Wu GWY Rampersaud R Reus VI Wolkowitz OM Mellon SH . Serum brain-derived neurotrophic factor, Val66Met polymorphism and open-label SSRI treatment response in Major Depressive Disorder. Psychoneuroendocrinology. (2024) 165:107045. doi: 10.1016/j.psyneuen.2024.107045, PMID: 38636352

[7] Dooley LN Kuhlman KR Robles TF Eisenberger NI Craske MG Bower JE . The role of inflammation in core features of depression: Insights from paradigms using exogenously-induced inflammation. Neurosci Biobehav Rev. (2018) 94:219–37. doi: 10.1016/j.neubiorev.2018.09.006, PMID: 30201219 PMC6192535

[8] Bekhbat M Li Z Mehta ND Treadway MT Lucido MJ Felger JC . Functional Connectivity in Reward Circuitry and Symptoms of Anhedonia as Therapeutic Targets in Depression with High Inflammation: Evidence from a Dopamine Challenge Study. Mol Psychiatry. (2022) 27:4113–21. doi: 10.1038/s41380-022-01715-3, PMID: 35927580 PMC9718669

[9] Hagenberg J BeCOME Study Group; OPTIMA Study Group Kahl LJ Stacey D Stelzhammer V Cooper JD . Dissecting Depression Symptoms: Multi-Omics Clustering Uncovers Immune-Related Subgroups and Cell-Type Specific Dysregulation. Brain Behav. Immun. (2025) 123:353–69. doi: 10.1016/j.bbi.2024.09.013, PMID: 39303816

[10] Felger JC Alagbe O Pace TW Woolwine BJ Hu F Raison CL . Early activation of p38 mitogen activated protein kinase is associated with interferon-alpha-induced depression and fatigue. Brain Behav Immun. (2011) 25:1094–8. doi: 10.1016/j.bbi.2011.02.015, PMID: 21356304 PMC3116018

[11] Eisenberger NI Inagaki TK Rameson LT Mashal NM Irwin MR . An fMRI study of cytokine-induced depressed mood and social pain: the role of sex differences. Neuroimage. (2009) 47:881–90. doi: 10.1016/j.neuroimage.2009.04.040, PMID: 19376240 PMC2733873

[12] Li P Zhao J Wei X Li H Yang Y Liu Y . Acupuncture May Play a Key Role in Anti-Depression through Various Mechanisms in Depression. Chin Med. (2024) 19:135. doi: 10.1186/s13020-024-00990-2, PMID: 39367470 PMC11451062

[13] Xu N He Y Wei YN Bai L Wang L . Possible Antidepressant Mechanism of Acupuncture: Targeting Neuroplasticity. Front Neurosci. (2025) 19:1512073. doi: 10.3389/fnins.2025.1512073, PMID: 40018358 PMC11865234

[14] Li J Wu X Yan S Wang L Tang Y Li K . Understanding the Antidepressant Mechanisms of Acupuncture: Targeting Hippocampal Neuroinflammation, Oxidative Stress, Neuroplasticity, and Apoptosis in CUMS Rats. Mol Neurobiol. (2025) 62:4221–36. doi: 10.1007/s12035-024-04550-5, PMID: 39422855 PMC11880061

[15] Jiang H Long X Wang Y Liu S Wang H Li X . Acupuncture Ameliorates Depression-Like Behaviors Through Modulating the Neuroinflammation Mediated by TLR4 Signaling Pathway in Rats Exposed to Chronic Restraint Stress. Mol Neurobiol. (2024) 61:2606–19. doi: 10.1007/s12035-023-03737-6, PMID: 37917302 PMC11043104

[16] Xu F Su Y Wang X Zhang T Xie T Wang Y . Olink Proteomics Analysis Uncovers Inflammatory Proteins in Patients with Different States of Bipolar Disorder. Int Immunopharmacol. (2024) 131:111816. doi: 10.1016/j.intimp.2024.111816, PMID: 38484669

[17] Zheng Y Cai X Wang D Li Y Zhang Y Liu X . Exploring the Relationship between Lipid Metabolism and Cognition in Individuals Living with Stable-Phase Schizophrenia: A Small Cross-Sectional Study Using Olink Proteomics Analysis. BMC Psychiatry. (2024) 24:593. doi: 10.1186/s12888-024-06054-x, PMID: 39227832 PMC11370234

[18] Yang L Cao M Tian J Li Y Zhang Y Wang Y . Identification of Plasma Inflammatory Markers of Adolescent Depression Using the Olink Proteomics Platform. J Inflamm Res. (2023) 16:4489–501. doi: 10.2147/JIR.S425780, PMID: 37849645 PMC10577244

[19] Li Z Chen L Huang Y Liu X Li H Wang Y . A Surface-Based Cross-Sectional fMRI Study on Brain Function Differences between Comorbid Mild or Moderate Depression and Insomnia. Front Neurosci. (2025) 19:1554287. doi: 10.3389/fnins.2025.1554287, PMID: 40433498 PMC12106362

[20] Vonderohe C Guthrie G Burrin DG . Fibroblast Growth Factor 19 Secretion and Function in Perinatal Development. Am J Physiol Gastrointest Liver Physiol (2023) 324:G190–5. doi: 10.1152/ajpgi.00208.2022, PMID: 36648144 PMC9942882

[21] Inagaki T Choi M Moschetta A Peng L Cummins CL McDonald JG . Fibroblast Growth Factor 15 Functions as an Enterohepatic Signal to Regulate Bile Acid Homeostasis. Cell Metab. (2005) 2:217–25. doi: 10.1016/j.cmet.2005.09.001, PMID: 16213224

[22] Gadaleta RM Garcia-Irigoyen O Cariello M Scialpi N Peres C Vetrano S . Fibroblast Growth Factor 19 Modulates Intestinal Microbiota and Inflammation in Presence of Farnesoid X Receptor. EBioMedicine. (2020) 54:102719. doi: 10.1016/j.ebiom.2020.102719, PMID: 32259714 PMC7136604

[23] Dolegowska K Marchelek-Mysliwiec M Nowosiad-Magda M Slawinski M Dolegowska B . FGF19 Subfamily Members: FGF19 and FGF21. J Physiol Biochem. (2019) 75:229–40. doi: 10.1007/s13105-019-00675-7, PMID: 30927227 PMC6611749

[24] Kan S Pi C Zhang L Ye F Li Z Li X . FGF19 Increases Mitochondrial Biogenesis and Fusion in Chondrocytes via the AMPKα-p38/MAPK Pathway. Cell Commun Signal. (2023) 21:55. doi: 10.1186/s12964-023-01069-5, PMID: 36915160 PMC10009974

[25] Ma J Yin X Cui K Wang J Li W Xu S . Mechanisms of Acupuncture in Treating Depression: A Review. Chin Med. (2025) 20:29. doi: 10.1186/s13020-025-01080-7, PMID: 40033393 PMC11877828

