# Supplementary material for: Olink proteomics identifies FGF-19 as a treatment-responsive inflammatory biomarker associated with acupuncture intervention in young females with mild depression
Source: Front Psychiatry. 2026 Feb 19;17:1743771. doi: 10.3389/fpsyt.2026.1743771 (PMC12960610; doi:10.3389/fpsyt.2026.1743771)
Supplement: Supplementary file 1 [file DataSheet1.pdf]

The FDR Adjusted MD vs ACU

Assay	p.valu	Adjusted_pval
CASP-8	0.01	0.42
FGF-5	0.01	0.42
IL2	0.02	0.42
FGF-19	0.02	0.42
CCL28	0.02	0.42
LAP TGF-beta-1	0.03	0.46
STAMBP	0.04	0.46
MCP-4	0.05	0.46
SLAMF1	0.05	0.46
4E-BP1	0.05	0.46
SIRT2	0.06	0.46
TWEAK	0.06	0.46
OSM	0.07	0.46
IL33	0.07	0.46
HGF	0.10	0.59
CX3CL1	0.11	0.60
CXCL5	0.12	0.60
MMP-1	0.12	0.60
VEGFA	0.12	0.60
CD8A	0.13	0.60
TNFB	0.14	0.60
CXCL6	0.14	0.60
MCP-2	0.16	0.60
MCP-3	0.16	0.60
MCP-1	0.16	0.60
CCL4	0.18	0.65
CST5	0.20	0.67
Flt3L	0.23	0.71
CCL11	0.24	0.71

DNER	0.24	0.71
CXCL1	0.24	0.71
SCF	0.25	0.72
CCL25	0.26	0.72
IL7	0.27	0.73
IL-15RA	0.28	0.73
CXCL11	0.32	0.77
TRAIL	0.33	0.77
uPA	0.33	0.77
FGF-21	0.36	0.77
IL18	0.36	0.77
EN-RAGE	0.36	0.77
IL-10RB	0.37	0.77
IL-20	0.38	0.77
CCL20	0.39	0.77
CSF-1	0.39	0.77
IFN-gamma	0.40	0.77
TGF-alpha	0.41	0.77
IL-2RB	0.41	0.77
IL-20RA	0.42	0.77
PD-L1	0.42	0.77
IL5	0.45	0.78
OPG	0.45	0.78
CXCL10	0.45	0.78
CDCP1	0.46	0.78
IL-10RA	0.47	0.78
CD5	0.51	0.83
IL4	0.51	0.83
ADA	0.53	0.83
LIF-R	0.53	0.83
Beta-NGF	0.56	0.85
CCL23	0.57	0.85

CCL3	0.59	0.86
NT-3	0.60	0.86
IL6	0.60	0.86
TNFRSF9	0.61	0.86
IL10	0.63	0.87
ARTN	0.64	0.87
LIF	0.65	0.87
CD6	0.65	0.87
TRANCE	0.67	0.87
IL-24	0.69	0.87
CXCL9	0.69	0.87
TNFSF14	0.70	0.87
AXIN1	0.71	0.87
IL-17A	0.71	0.87
NRTN	0.72	0.87
IL13	0.73	0.87
CD40	0.74	0.87
CD244	0.78	0.90
IL-12B	0.78	0.90
TNF	0.80	0.91
FGF-23	0.84	0.93
ST1A1	0.84	0.93
IL-1 alpha	0.87	0.95
IL8	0.89	0.95
IL-22 RA1	0.89	0.95
GDNF	0.90	0.95
IL-18R1	0.92	0.96
CCL19	0.96	0.99
TSLP	0.98	0.99
IL-17C	0.99	0.99
MMP-10	0.99	0.99

The FDR Adjusted HC vs MD

Assay	p.valu	Adjusted_pval
SIRT2	0.00	0.01
AXIN1	0.00	0.04
4E-BP1	0.00	0.05
DNER	0.00	0.05
CASP-8	0.00	0.05
STAMBP	0.00	0.05
CCL11	0.01	0.12
CD5	0.01	0.12
ADA	0.01	0.12
CD244	0.02	0.20
FGF-19	0.03	0.21
TWEAK	0.03	0.22
NRTN	0.03	0.24
IL-17A	0.04	0.25
IL13	0.05	0.30
ST1A1	0.05	0.30
TNFRSF9	0.06	0.35
CX3CL1	0.07	0.36
TNFB	0.08	0.36
LAP TGF-beta-1	0.09	0.40
IL-10RA	0.09	0.40
FGF-21	0.10	0.40
CCL20	0.10	0.40
IL-2RB	0.11	0.40
CCL28	0.11	0.40
TGF-alpha	0.12	0.43
LIF-R	0.15	0.50
CXCL5	0.15	0.50

PD-L1	0.16	0.51
CCL3	0.18	0.54
IL-1 alpha	0.18	0.54
SCF	0.19	0.55
IL-12B	0.22	0.60
IL-17C	0.22	0.60
TNF	0.24	0.63
CD6	0.25	0.63
CCL19	0.26	0.63
CXCL6	0.26	0.63
NT-3	0.27	0.63
IFN-gamma	0.29	0.63
Flt3L	0.30	0.63
HGF	0.30	0.63
TSLP	0.30	0.63
FGF-5	0.32	0.63
MMP-1	0.32	0.63
IL-24	0.32	0.63
IL-18R1	0.32	0.63
IL2	0.34	0.64
VEGFA	0.34	0.64
IL-20RA	0.35	0.64
IL-20	0.37	0.66
IL4	0.37	0.66
IL6	0.38	0.66
MCP-2	0.39	0.67
IL10	0.42	0.70
IL8	0.43	0.70
MMP-10	0.44	0.70
CSF-1	0.44	0.70
CCL4	0.45	0.70
LIF	0.46	0.70

uPA	0.46	0.70
Beta-NGF	0.49	0.72
MCP-4	0.49	0.72
IL7	0.52	0.73
IL5	0.52	0.73
CCL23	0.53	0.74
CXCL1	0.54	0.74
TRAIL	0.56	0.76
FGF-23	0.57	0.76
CST5	0.59	0.77
GDNF	0.60	0.77
CCL25	0.60	0.77
CD40	0.61	0.77
IL-10RB	0.65	0.81
CD8A	0.69	0.84
MCP-1	0.69	0.84
CDCP1	0.72	0.86
CXCL10	0.74	0.87
IL18	0.74	0.87
OSM	0.77	0.88
CXCL9	0.78	0.88
SLAMF1	0.84	0.93
IL33	0.87	0.93
ARTN	0.87	0.93
IL-15RA	0.87	0.93
TRANCE	0.88	0.93
OPG	0.88	0.93
IL-22 RA1	0.93	0.97
TNFSF14	0.95	0.97
CXCL11	0.95	0.97
MCP-3	0.96	0.97
EN-RAGE	0.99	0.99

MDVSHC_auc_detailed_summary

Biomarker	AUC	AUC_95CI	Lower_95CI	Upper_95CI	AUC_Interpretation
AXIN1	0.9200	0.9200 [0.7798-1.0000]	0.7798	1.0000	Excellent
SIRT2	0.9200	0.9200 [0.7500-1.0000]	0.7500	1.0000	Excellent
DNER	0.8700	0.8700 [0.6900-1.0000]	0.6900	1.0000	Good
4E-BP1	0.8600	0.8600 [0.6500-1.0000]	0.6500	1.0000	Good
CASP-8	0.8500	0.8500 [0.6500-1.0000]	0.6500	1.0000	Good
STAMBP	0.8400	0.8400 [0.6200-1.0000]	0.6200	1.0000	Good
CD5	0.8300	0.8300 [0.6100-0.9702]	0.6100	0.9702	Good
CCL11	0.8200	0.8200 [0.6000-0.9800]	0.6000	0.9800	Good
CD244	0.8000	0.8000 [0.5600-1.0000]	0.5600	1.0000	Good
NRTN	0.8000	0.8000 [0.5800-0.9600]	0.5800	0.9600	Good
TWEAK	0.8000	0.8000 [0.5900-0.9700]	0.5900	0.9700	Good
ADA	0.8000	0.8000 [0.5800-0.9700]	0.5800	0.9700	Good
IL-17A	0.7900	0.7900 [0.5500-0.9700]	0.5500	0.9700	Fair
IL13	0.7900	0.7900 [0.5700-0.9600]	0.5700	0.9600	Fair
FGF-19	0.7900	0.7900 [0.5500-0.9800]	0.5500	0.9800	Fair
CCL20	0.7700	0.7700 [0.5300-0.9500]	0.5300	0.9500	Fair
FGF-21	0.7600	0.7600 [0.5100-0.9600]	0.5100	0.9600	Fair
CX3CL1	0.7600	0.7600 [0.5298-0.9500]	0.5298	0.9500	Fair
TNFB	0.7400	0.7400 [0.4998-0.9600]	0.4998	0.9600	Fair
IL-2RB	0.7300	0.7300 [0.4800-0.9400]	0.4800	0.9400	Fair
LIF-R	0.7200	0.7200 [0.4700-0.9200]	0.4700	0.9200	Fair
CCL28	0.7200	0.7200 [0.4600-0.9202]	0.4600	0.9202	Fair
TNFRSF9	0.7200	0.7200 [0.4500-0.9400]	0.4500	0.9400	Fair
LAP TGF-beta-1	0.7100	0.7100 [0.4700-0.9300]	0.4700	0.9300	Fair
ST1A1	0.7100	0.7100 [0.4500-0.9200]	0.4500	0.9200	Fair
SCF	0.7000	0.7000 [0.4400-0.9200]	0.4400	0.9200	Fair
CXCL5	0.7000	0.7000 [0.4300-0.9200]	0.4300	0.9200	Fair
CCL3	0.6900	0.6900 [0.4500-0.9100]	0.4500	0.9100	Poor

TGF-alpha	0.6900	0.6900	[0.4400-0.9100]	0.4400	0.9100	Poor
CXCL10	0.6800	0.6800	[0.4200-0.9000]	0.4200	0.9000	Poor
LIF	0.6800	0.6800	[0.4200-0.9100]	0.4200	0.9100	Poor
IL-17C	0.6700	0.6700	[0.4100-0.9000]	0.4100	0.9000	Poor
MCP-4	0.6700	0.6700	[0.3998-0.9000]	0.3998	0.9000	Poor
MMP-1	0.6700	0.6700	[0.4100-0.8900]	0.4100	0.8900	Poor
CXCL1	0.6600	0.6600	[0.3898-0.9100]	0.3898	0.9100	Poor
CCL19	0.6500	0.6500	[0.3798-0.8700]	0.3798	0.8700	Poor
TNF	0.6500	0.6500	[0.3798-0.8800]	0.3798	0.8800	Poor
MCP-3	0.6400	0.6400	[0.3600-0.9000]	0.3600	0.9000	Poor
IL-1 alpha	0.6400	0.6400	[0.3500-0.8700]	0.3500	0.8700	Poor
IL-10RA	0.6400	0.6400	[0.3798-0.8700]	0.3798	0.8700	Poor
IL-12B	0.6400	0.6400	[0.3700-0.8700]	0.3700	0.8700	Poor
IL4	0.6400	0.6400	[0.3600-0.9000]	0.3600	0.9000	Poor
NT-3	0.6400	0.6400	[0.3700-0.8800]	0.3700	0.8800	Poor
IL2	0.6300	0.6300	[0.3600-0.8600]	0.3600	0.8600	Poor
PD-L1	0.6300	0.6300	[0.3600-0.8600]	0.3600	0.8600	Poor
CD6	0.6200	0.6200	[0.3500-0.8600]	0.3500	0.8600	Poor
IL5	0.6200	0.6200	[0.3500-0.8600]	0.3500	0.8600	Poor
CSF-1	0.6200	0.6200	[0.3600-0.8900]	0.3600	0.8900	Poor
CXCL6	0.6100	0.6100	[0.3400-0.8502]	0.3400	0.8502	Poor
IFN-gamma	0.6100	0.6100	[0.3400-0.8600]	0.3400	0.8600	Poor
VEGFA	0.6000	0.6000	[0.3400-0.8500]	0.3400	0.8500	Poor
Beta-NGF	0.6000	0.6000	[0.3300-0.8500]	0.3300	0.8500	Poor
IL10	0.6000	0.6000	[0.3200-0.8400]	0.3200	0.8400	Poor
CCL23	0.6000	0.6000	[0.3300-0.8500]	0.3300	0.8500	Poor
Flt3L	0.6000	0.6000	[0.3200-0.8600]	0.3200	0.8600	Poor
CD40	0.6000	0.6000	[0.3300-0.8500]	0.3300	0.8500	Poor
MCP-1	0.5800	0.5800	[0.3000-0.8200]	0.3000	0.8200	Fail
IL-15RA	0.5800	0.5800	[0.3200-0.8400]	0.3200	0.8400	Fail
MMP-10	0.5800	0.5800	[0.3200-0.8300]	0.3200	0.8300	Fail
IL8	0.5800	0.5800	[0.3000-0.8400]	0.3000	0.8400	Fail
IL6	0.5800	0.5800	[0.3000-0.8400]	0.3000	0.8400	Fail

FGF-5	0.5800	0.5800	[0.3100-0.8202]	0.3100	0.8202	Fail
IL-20	0.5700	0.5700	[0.3000-0.8100]	0.3000	0.8100	Fail
TRAIL	0.5700	0.5700	[0.2898-0.8300]	0.2898	0.8300	Fail
CCL4	0.5600	0.5600	[0.2700-0.8000]	0.2700	0.8000	Fail
TRANCE	0.5600	0.5600	[0.2900-0.8100]	0.2900	0.8100	Fail
IL-24	0.5600	0.5600	[0.3000-0.8300]	0.3000	0.8300	Fail
CXCL9	0.5500	0.5500	[0.2900-0.8200]	0.2900	0.8200	Fail
TNFSF14	0.5500	0.5500	[0.2900-0.8200]	0.2900	0.8200	Fail
IL-10RB	0.5500	0.5500	[0.2900-0.8002]	0.2900	0.8002	Fail
OPG	0.5500	0.5500	[0.2900-0.8200]	0.2900	0.8200	Fail
CDCP1	0.5400	0.5400	[0.2800-0.7902]	0.2800	0.7902	Fail
CST5	0.5400	0.5400	[0.2798-0.7802]	0.2798	0.7802	Fail
IL-22 RA1	0.5400	0.5400	[0.2700-0.8302]	0.2700	0.8302	Fail
GDNF	0.5300	0.5300	[0.2700-0.7800]	0.2700	0.7800	Fail
EN-RAGE	0.5300	0.5300	[0.2600-0.7800]	0.2600	0.7800	Fail
MCP-2	0.5300	0.5300	[0.2600-0.7900]	0.2600	0.7900	Fail
IL7	0.5200	0.5200	[0.2500-0.7900]	0.2500	0.7900	Fail
IL18	0.5200	0.5200	[0.2498-0.7800]	0.2498	0.7800	Fail
CD8A	0.5200	0.5200	[0.2698-0.7800]	0.2698	0.7800	Fail
CXCL11	0.5100	0.5100	[0.2500-0.7900]	0.2500	0.7900	Fail
SLAMF1	0.4900	0.4900	[0.2200-0.7500]	0.2200	0.7500	Fail
ARTN	0.4900	0.4900	[0.2298-0.7600]	0.2298	0.7600	Fail
IL-20RA	0.4800	0.4800	[0.2400-0.7400]	0.2400	0.7400	Fail
IL33	0.4800	0.4800	[0.2100-0.7400]	0.2100	0.7400	Fail
FGF-23	0.4700	0.4700	[0.2000-0.7300]	0.2000	0.7300	Fail
uPA	0.4600	0.4600	[0.2100-0.7200]	0.2100	0.7200	Fail
OSM	0.4500	0.4500	[0.2100-0.7200]	0.2100	0.7200	Fail
TSLP	0.4500	0.4500	[0.2100-0.7102]	0.2100	0.7102	Fail
CCL25	0.4400	0.4400	[0.1800-0.7000]	0.1800	0.7000	Fail
HGF	0.4000	0.4000	[0.1600-0.6700]	0.1600	0.6700	Fail
IL-18R1	0.3900	0.3900	[0.1600-0.6600]	0.1600	0.6600	Fail

ACUVSMD_auc_detailed_summary

Biomarker	AUC	AUC_95CI	Lower_95CI	Upper_95CI	AUC_Interpretation
IL2	0.8000	0.8000 [0.5700-0.9600]	0.5700	0.9600	Good
FGF-19	0.8000	0.8000 [0.5698-0.9800]	0.5698	0.9800	Good
FGF-5	0.7600	0.7600 [0.5200-0.9400]	0.5200	0.9400	Fair
CASP-8	0.7400	0.7400 [0.4900-0.9600]	0.4900	0.9600	Fair
4E-BP1	0.7300	0.7300 [0.4900-0.9200]	0.4900	0.9200	Fair
SIRT2	0.7100	0.7100 [0.4500-0.9100]	0.4500	0.9100	Fair
IL33	0.7000	0.7000 [0.4400-0.9300]	0.4400	0.9300	Fair
STAMBP	0.7000	0.7000 [0.4400-0.9200]	0.4400	0.9200	Fair
CXCL9	0.6800	0.6800 [0.4100-0.9100]	0.4100	0.9100	Poor
CCL28	0.6800	0.6800 [0.4200-0.8900]	0.4200	0.8900	Poor
LAP TGF-beta-1	0.6700	0.6700 [0.4100-0.9000]	0.4100	0.9000	Poor
CXCL6	0.6700	0.6700 [0.4198-0.8902]	0.4198	0.8902	Poor
IL-20	0.6600	0.6600 [0.3900-0.9000]	0.3900	0.9000	Poor
HGF	0.6500	0.6500 [0.3800-0.8800]	0.3800	0.8800	Poor
TNFB	0.6500	0.6500 [0.3800-0.8800]	0.3800	0.8800	Poor
TRAIL	0.6400	0.6400 [0.3600-0.9000]	0.3600	0.9000	Poor
IL-15RA	0.6400	0.6400 [0.3600-0.8800]	0.3600	0.8800	Poor
Flt3L	0.6400	0.6400 [0.3800-0.8800]	0.3800	0.8800	Poor
OPG	0.6300	0.6300 [0.3600-0.8900]	0.3600	0.8900	Poor
OSM	0.6300	0.6300 [0.3600-0.8700]	0.3600	0.8700	Poor
MCP-3	0.6200	0.6200 [0.3600-0.8400]	0.3600	0.8400	Poor
uPA	0.6200	0.6200 [0.3500-0.8600]	0.3500	0.8600	Poor
SLAMF1	0.6200	0.6200 [0.3600-0.8700]	0.3600	0.8700	Poor
TGF-alpha	0.6200	0.6200 [0.3500-0.8700]	0.3500	0.8700	Poor
CCL11	0.6200	0.6200 [0.3600-0.8800]	0.3600	0.8800	Poor
CD5	0.6200	0.6200 [0.3700-0.8502]	0.3700	0.8502	Poor
CX3CL1	0.6200	0.6200 [0.3500-0.8700]	0.3500	0.8700	Poor

TWEAK	0.6200	0.6200	[0.3600-0.8602]	0.3600	0.8602	Poor
IL6	0.6100	0.6100	[0.3600-0.8500]	0.3600	0.8500	Poor
CST5	0.6100	0.6100	[0.3400-0.8400]	0.3400	0.8400	Poor
PD-L1	0.6000	0.6000	[0.3300-0.8600]	0.3300	0.8600	Poor
LIF-R	0.5900	0.5900	[0.3200-0.8400]	0.3200	0.8400	Fail
VEGFA	0.5900	0.5900	[0.3100-0.8300]	0.3100	0.8300	Fail
MCP-1	0.5900	0.5900	[0.3200-0.8500]	0.3200	0.8500	Fail
MCP-4	0.5900	0.5900	[0.3398-0.8500]	0.3398	0.8500	Fail
NT-3	0.5900	0.5900	[0.3000-0.8200]	0.3000	0.8200	Fail
IL5	0.5900	0.5900	[0.3200-0.8500]	0.3200	0.8500	Fail
FGF-21	0.5800	0.5800	[0.3100-0.8300]	0.3100	0.8300	Fail
CDCP1	0.5800	0.5800	[0.3200-0.8300]	0.3200	0.8300	Fail
IL-2RB	0.5800	0.5800	[0.3100-0.8400]	0.3100	0.8400	Fail
TNFSF14	0.5800	0.5800	[0.3200-0.8300]	0.3200	0.8300	Fail
Beta-NGF	0.5800	0.5800	[0.3100-0.8400]	0.3100	0.8400	Fail
TNFRSF9	0.5800	0.5800	[0.2900-0.8600]	0.2900	0.8600	Fail
CCL4	0.5700	0.5700	[0.3100-0.8400]	0.3100	0.8400	Fail
ARTN	0.5700	0.5700	[0.3100-0.8200]	0.3100	0.8200	Fail
CCL25	0.5700	0.5700	[0.3100-0.8300]	0.3100	0.8300	Fail
IFN-gamma	0.5700	0.5700	[0.3100-0.8200]	0.3100	0.8200	Fail
CCL19	0.5600	0.5600	[0.2900-0.8300]	0.2900	0.8300	Fail
CCL23	0.5600	0.5600	[0.3000-0.8100]	0.3000	0.8100	Fail
CXCL10	0.5600	0.5600	[0.3100-0.8100]	0.3100	0.8100	Fail
DNER	0.5600	0.5600	[0.2800-0.8100]	0.2800	0.8100	Fail
CD8A	0.5600	0.5600	[0.3000-0.8300]	0.3000	0.8300	Fail
IL8	0.5600	0.5600	[0.2900-0.8200]	0.2900	0.8200	Fail
ST1A1	0.5600	0.5600	[0.2900-0.8200]	0.2900	0.8200	Fail
AXIN1	0.5500	0.5500	[0.2800-0.8100]	0.2800	0.8100	Fail
TSLP	0.5500	0.5500	[0.2900-0.7900]	0.2900	0.7900	Fail
MMP-1	0.5500	0.5500	[0.2800-0.8200]	0.2800	0.8200	Fail
IL13	0.5500	0.5500	[0.2898-0.8000]	0.2898	0.8000	Fail
IL10	0.5500	0.5500	[0.2800-0.8100]	0.2800	0.8100	Fail
LIF	0.5500	0.5500	[0.2900-0.8100]	0.2900	0.8100	Fail

NRTN	0.5500	0.5500	[0.2800-0.8002]	0.2800	0.8002	Fail
TNF	0.5500	0.5500	[0.2800-0.8202]	0.2800	0.8202	Fail
IL-10RB	0.5500	0.5500	[0.2800-0.8100]	0.2800	0.8100	Fail
IL-12B	0.5500	0.5500	[0.2800-0.8300]	0.2800	0.8300	Fail
IL4	0.5500	0.5500	[0.2800-0.8000]	0.2800	0.8000	Fail
CD244	0.5400	0.5400	[0.2800-0.8000]	0.2800	0.8000	Fail
IL-17A	0.5400	0.5400	[0.2700-0.8000]	0.2700	0.8000	Fail
IL-1 alpha	0.5400	0.5400	[0.2600-0.8000]	0.2600	0.8000	Fail
EN-RAGE	0.5400	0.5400	[0.2800-0.8000]	0.2800	0.8000	Fail
CD6	0.5300	0.5300	[0.2600-0.7800]	0.2600	0.7800	Fail
IL-10RA	0.5300	0.5300	[0.2700-0.7900]	0.2700	0.7900	Fail
IL-22 RA1	0.5300	0.5300	[0.2500-0.7900]	0.2500	0.7900	Fail
CCL20	0.5300	0.5300	[0.2800-0.7900]	0.2800	0.7900	Fail
CCL3	0.5200	0.5200	[0.2600-0.7900]	0.2600	0.7900	Fail
ADA	0.5200	0.5200	[0.2498-0.7800]	0.2498	0.7800	Fail
IL18	0.5100	0.5100	[0.2500-0.7800]	0.2500	0.7800	Fail
IL-24	0.5100	0.5100	[0.2500-0.7800]	0.2500	0.7800	Fail
MMP-10	0.5100	0.5100	[0.2600-0.7700]	0.2600	0.7700	Fail
GDNF	0.5000	0.5000	[0.2400-0.7700]	0.2400	0.7700	Fail
IL-17C	0.5000	0.5000	[0.2300-0.7800]	0.2300	0.7800	Fail
FGF-23	0.5000	0.5000	[0.2400-0.7700]	0.2400	0.7700	Fail
IL-18R1	0.5000	0.5000	[0.2400-0.7700]	0.2400	0.7700	Fail
MCP-2	0.4900	0.4900	[0.2400-0.7600]	0.2400	0.7600	Fail
CSF-1	0.4900	0.4900	[0.2398-0.7700]	0.2398	0.7700	Fail
TRANCE	0.4800	0.4800	[0.2200-0.7400]	0.2200	0.7400	Fail
IL7	0.4700	0.4700	[0.2100-0.7300]	0.2100	0.7300	Fail
IL-20RA	0.4700	0.4700	[0.2200-0.7400]	0.2200	0.7400	Fail
CXCL1	0.4600	0.4600	[0.2100-0.7302]	0.2100	0.7302	Fail
SCF	0.4600	0.4600	[0.1998-0.7200]	0.1998	0.7200	Fail
CD40	0.4500	0.4500	[0.1900-0.7200]	0.1900	0.7200	Fail
CXCL5	0.4200	0.4200	[0.1800-0.6900]	0.1800	0.6900	Fail
CXCL11	0.4100	0.4100	[0.1600-0.7000]	0.1600	0.7000	Fail
